# Modification of Renewable Cardanol onto Carbon Fiber for the Improved Interfacial Properties of Advanced Polymer Composites

**DOI:** 10.3390/polym12010045

**Published:** 2019-12-28

**Authors:** Yawen Zheng, Lei Chen, Xiaoyun Wang, Guangshun Wu

**Affiliations:** 1 School of Chemistry and Materials Science, Ludong University, Yantai 264025, China; zheng1ya2wen3@163.com (Y.Z.); wxyangao@163.com (X.W.); 2 College of Textiles and Garments, Southwest University, Chongqing 400715, China; raychen@swu.edu.cn

**Keywords:** carbon fibers, polymer-based composites, fiber–matrix interface, cardanol, surface grafting

## Abstract

A facile in situ polymerization was developed for grafting renewable cardanol onto the carbon fiber (CF) surfaces to strengthen the fiber–matrix interface. CFs were chemically modified with hydroxyl groups by using an aryl diazonium reaction, and then copolymerized in situ with hexachlorocyclotriphosphazene (HCCP) and cardanol to build cardanol-modified fibers (CF-cardanol). The cardanol molecules were successfully introduced, as confirmed using Raman spectra and X-ray photoelectron spectroscopy (XPS); the cardanol molecules were found to increase the surface roughness, energy, interfacial wettability, and activity with the matrix resin. As a result, the interlaminar shear strength (ILSS) of CF-cardanol composites increased from 48.2 to 68.13 MPa. In addition, the anti-hydrothermal ageing properties of the modified composites were significantly increased. The reinforcing mechanisms of the fiber–matrix interface were also studied.

## 1. Introduction

Carbon-fiber-reinforced polymer composites have attracted scientific attention from many groups aiming for scientific research and industrial utilization, and are widely used as novel engineering materials in many fields because of their high tensile strength, light weight, and outstanding environmental stability [1,2,3,4,5,6]. Nevertheless, the practical application of carbon fiber (CF) composites has been limited because of the fibers’ inert and smooth surfaces, as well as the poor quality of the resultant fiber–matrix interface [7,8,9]. Hence, modifying the CF surfaces to create good interfacial properties can be crucial for increasing the composite’s overall performances [10,11]. Recently, many approaches have been proposed for modifying carbon fiber surfaces to promote the interface properties of composites, such as coating or sizing, high-energy radiation, oxidation, chemical modification, and so on [12,13,14,15,16,17,18,19,20,21]. Among these approaches, chemical modification is considered to be one of the most effective approaches due to its simplicity and controllability without high energy.

The chemical grafting of polymers or micromolecules onto the surface of CFs can yield a controlled, ordered, and hierarchical reinforcement by effectively reinforcing the fiber–matrix interface [22,23]. Ma et al. [24] grafted branched polyethyleneimine (PEI) onto carbon fiber surfaces using supercritical fluids or chemical modification, and the interfacial and mechanical properties were improved significantly. The in situ grafting of hyperbranched polyglycerol onto CF surfaces using anionic ring-opening polymerization for enhancing interfacial quality was proposed [25]. Those modifications either involved a strong acid treatment to build the active site, expensive materials, or high energy consumption. Interfacial behaviors of CF-reinforced matrix composites are mainly affected by many important factors, such as interfacial wettability, the introduced mechanical interlocking, and the formed chemical bonding at the interface [26]. In a word, the formed chemical bonding between CFs and the matrix resin promotes the largest share of composite interface improvements.

Cardanol, which is extracted from cashew nut shell liquid using a double vacuum distillation technique, is known as a significant agricultural renewable resource [27,28,29,30]. Cardanol, with a reactive phenolic moiety and an unsaturated C15 hydrocarbon chain, can easily form cross-linked structures under a thermal curing treatment, which is viewed as a robust platform for further grafting for multifunctional applications in the fields of coatings, biocomposites, curing agents, and antioxidants owing to its sustainability, low cost, large availability, and bactericidal properties [31,32]. We believe that this is the first report of a new hierarchical reinforcement that has been surface-modified with cardanol for changing the surface/interface properties. Herein, we developed a high-efficiency strategy for functionalizing CFs with renewable cardanol and studied the interfacial behaviors and the mechanisms of reinforcing, as well as toughening the interface for unsaturated polyester resin (UPR) composites. The active sites were built onto CF surfaces by using the diazonium technique, avoiding a strong acid oxidation treatment. As an agricultural renewable resource, cardanol is cost-effective, highlighting the potential of the strategy for practical applications. Moreover, the chemical bonding easily formed at the interface between CF-cardanol and UPR during the curing process, which played the most important role in enhancing the composite’s interfacial properties. The preparation and characterization of CFs functionalized with cardanol and the resultant composites were fully studied, as discussed in this work.

## 2. Materials and Methods

### 2.1. Materials

Long polyacrylonitrile (PAN)-based carbon fibers (CFs) (3 × 10^3^ single filaments per tow, diameter of 6–7 μm, density of 1.76 g·cm^−3^, tensile strength of 3500 MPa) were obtained from Toray Industries, Inc (Tokyo, Japan). Prior to use, fiber-sizing agents of the as-received CF (sized CF) were removed as described in our previous paper [33]. AROPOL MR13006 polyester, as well as the agent LP4016, were received from Ashland Inc. (Kentucky, KY, USA). Tert-Butyl peroxybenzoate (TBPB), used as the initiator, was provided by ACROS ORGANICS Inc (Beijing, China). Cardanol and hexachlorocyclotriphosphazene (HCCP) were supplied by Aladdin (Shanghai, China) and Energy Chemical Reagent Co., Ltd. (Shanghai, China), respectively. All solvents and other reagents were received from Tianjin Bodi Organic Chemicals Co. Ltd. (Tianjin, China).

### 2.2. Fiber Surface Modification

**Figure 1:**
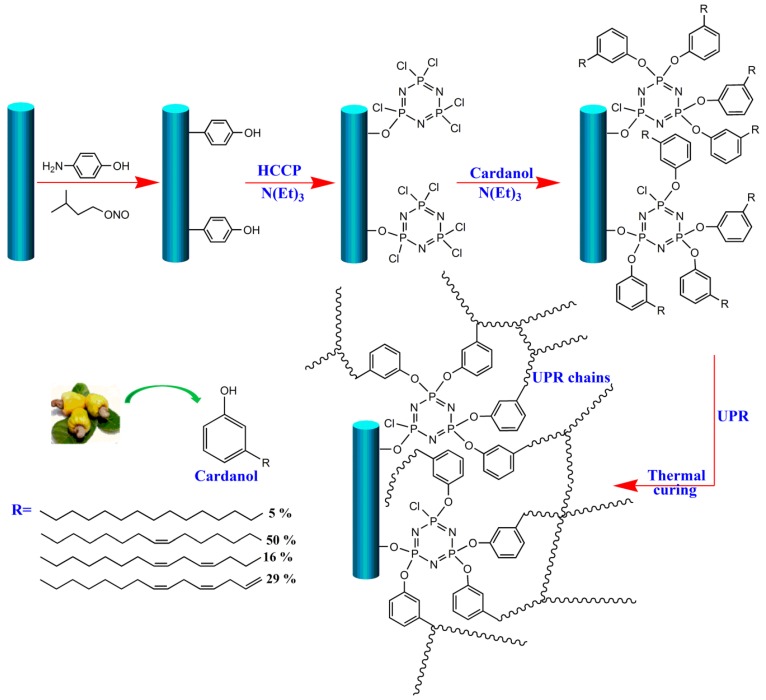
Schematic illustration of the preparation procedure of CF-cardanol and the interfacial reaction between modified CFs and the matrix. HCCP: hexachlorocyclotriphosphazene, UPR: unsaturated polyester resin.

A glass frame with untreated CFs was treated with 0.5 mL of isopentyl nitrite and 44-aminopheno-phenol in 50 mL of distilled water, and then stirred mechanically at 353 K for 12 h with the aim of introducing polar hydroxyl groups to change the fiber surface and provide the reaction sites for further grafting. After the reaction, the product was fully washed using massive *N,N*-Dimethylformamide (DMF) and distilled water to remove unreacted molecules and impurities to obtain CF-OH. Subsequently, the obtained fibers were added into the mixed solution containing 50 mL dimethyl sulfoxide (DMSO), 5.4 mmol HCCP, and 4.5 mL triethylamine, and allowed to react at 323 K for 2 h. After that, cardanol molecules were quickly put into the above mixed solution to react via stirring for 8 h. At the end of the reaction, the modified fibers (denoted as CF-cardanol) were washed in DMSO and acetone using sonication several times to remove impurities. A schematic illustration of the whole functionalized procedure of CF-cardanol is shown in Figure 1.

### 2.3. Preparation of CF/UPR Composites

The unidirectional prepreg of the fiber and matrix used to manufacture CF/UPR composites was first prepared as described in great detail in a previous work [34]. Then, the prepreg was added into a mold for preparing unidirectional composites on a hot press machine based on a compression molding method. CF/UPR composites were prepared using a thermal curing treatment according to the following conditions: 353 K for about 1 h, 373 K with a pressure of 10 MPa for 1 h, and 413 K under 10 MPa for 1 h. Finally, the mold was cooled naturally while maintaining the pressure. The fiber contents of the composites can be easily obtained based on the following equation:

(1) $$Content=\frac{nLX}{m}$$

where *X* and *L* are the linear density of carbon fiber (g/mm) and the length of the sample (mm), respectively. *N* and *m* are the total numbers of carbon fibers and the weight of the sample (g), respectively. The fiber contents of composites were controlled at about 70% *w*/*w*. The measured samples with the dimensions of 2 mm × 20 mm × 6 mm and 2 mm × 55 mm × 6 mm were fit for interlaminar shear strength (ILSS) and impact toughness testing, respectively.

### 2.4. Characterization Techniques

The elemental composition of untreated and modified fibers were characterized using Raman spectrometer (HR Evolution, Horiba, France) using the excitation wavelength of 532 nm. Furthermore, the surface structure of different fibers was measured using X-ray photoelectron spectroscopy (XPS, ESCALAB 220i-XL, VG, UK), which was analyzed with the monochromatic Al Kα source (1486.6 eV) and the base pressure (2 × 10^−9^ mbar).

Surface microstructures of CFs with and without treatment, as well as untreated and modified composite cracked sections after ILSS testing, were examined using scanning electron microscopy (SEM, Quanta 200FEG, Hitachi Instrument, Inc., Tokyo, Japan). It was performed with an accelerating voltage of 20 kV. All the testing fibers and composites needed to be sputtered with thin gold layers of about 2 nm before SEM testing.

The interfacial wettability between different CFs and the matrix resin was examined via the fiber surface energy, which was measured on a dynamic contact angle meter (DCAT21, Data Physics Instruments, Stuttgart, Germany). The surface energy and its components were obtained via immersing the fibers into two testing liquids (deionised water and diiodomethane) to record fiber contact angles for the surface energy calculations, which were easily found using the following equations:

(2) $$\gamma_{l}(1+\cos\theta )=2{\left(\gamma_{l}^{p}\gamma_{f}^{p}\right)}^{1/2}+2{\left(\gamma_{l}^{d}\gamma_{f}^{d}\right)}^{1/2},$$

(3) $$\gamma_{f}=\gamma_{f}^{p}+\gamma_{f}^{d},$$

where $\gamma_{l}$, $\gamma_{l}^{p}$, and $\gamma_{l}^{d}$ are surface tension of the testing liquids, the polar component, and the dispersive component, respectively.

A universal testing machine (WD-1, Changchun, China) was used to examine the ILSS values of the untreated and modified composites. ILSS values of the composites were calculated using:

(4) $$ILSS=\frac{3Pb}{4bh},$$

where *P_b_* is the maximum breaking load (N), *b* and *h* are the width (mm) and thickness (mm) of the sample, respectively.

Untreated CF, CF-OH, and CF-cardanol-reinforced UPR composites were added into boiling water (373 K) and then treated under this condition for 48 h. Finally, the hydrothermal aging resistances of the resulting composites were tested by comparing the changes of the composites’ ILSS values with and without the treatment.

## 3. Results

### 3.1. Surface Composition and Structure of CFs

**Figure 2:**
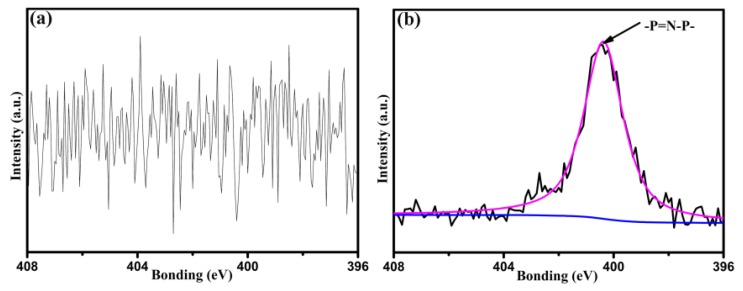
High-resolution XPS N 1s curve fitting for the CFs: (**a**) untreated CF and (**b**) CF-cardanol.

XPS is useful to examine surface composition of materials. Table 1 presents the surface chemical composition of untreated CF, CF-OH, and CF-cardanol. Untreated CF and CF-OH were composed of carbon, oxygen, nitrogen, and a small amount of chlorine. However, their surfaces showed the distinct contents of carbon and oxygen. The increase of oxygen content and the decrease of carbon content, compared with those of the untreated fibers, may be related to the introduced phenolic hydroxyl groups by the aryl diazonium reaction. As a result, the atomic O/C ratios of the CF-OH surfaces improved from 0.21 to 0.31. The introduced phenolic hydroxyl groups can be used as an efficient and robust platform to modify HCCP and cardanol onto the fiber surface. After being introduced with cardanol, a significant phosphorus component of 3.61% appeared on the surface of CF-cardanol, and the nitrogen content significantly improved from 1.21% to 6.49% owing to the modified fibers of HCCP and cardanol copolymerization. The N 1s spectra of untreated CF and CF-cardanol are shown in Figure 2. From Figure 2a, there were no peaks observed on the N 1s spectrum of untreated CF. However, after being grafted by cardanol, the N 1s spectrum of CF-cardanol could be fitted to one peak, which was assigned to –P=N–P– arising from the copolymerized structure with hexachlorocyclotriphosphazene (HCCP) and cardanol, indicating the successful polymerization and the grafting of cardanol onto the fiber surface.

**Figure 3:**
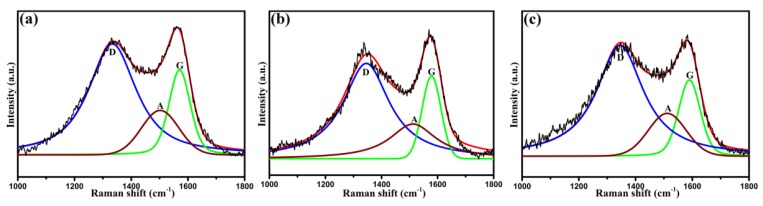
Raman spectra and curve fitting for the CFs: (**a**) untreated CF, (**b**) CF-OH, and (**c**) CF-cardanol.

Raman spectroscopy was used to analyze the surface structures of untreated CF, CF-OH, and CF-cardanol, as shown in Figure 3. From Figure 3, untreated and modified fibers mainly had three large bands, which were the D band (about 1345 cm^−1^), A band (about 1500 cm^−1^), and G band (about 1590 cm^−1^). The D band is related to defects in the carbonaceous structure, while the G band is due to ordered graphitic structures [35]. The A band represents amorphous forms of carbon or interstitial defects [35,36]. The integral intensity ratio of the D band to the G band (R, *I_D_*/*I_G_*) is considered as a measure of the degree of defects in the carbonaceous materials. The *I_D_*/*I_G_* value of untreated CF was 3.49, while the *I_D_*/*I_G_* value of CF-OH increased to 3.84, indicating that the aryl diazonium treatment disrupted the graphite minicrystal ordered structure and thus obtained a higher disordered degree. It also demonstrates the successful modification of phenol groups. After being introduced via cardanol, the *I_D_*/*I_G_* of CF-cardanol decreased slightly from 3.84 to 3.83, confirming that the copolymerized in situ with HCCP and cardanol for preparing cardanol modified fibers using phenol groups was an efficient platform that did not further change the fiber surface’s ordered degree.

### 3.2. Surface Microstructures of CFs

**Figure 4:**
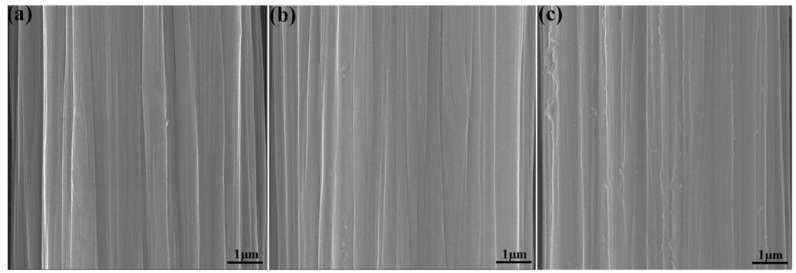
SEM images of different CF surfaces: (**a**) untreated CF, (**b**) CF-OH, and (**c**) CF-cardanol.

Figure 4 presents the SEM images of the surface microstructures of untreated CF, CF-OH, and CF-cardanol. The smooth and inert surface of untreated CF (Figure 4a) is observed. For CF-OH (Figure 4b), CF-OH had a similar microstructure surface compared to that of untreated CF, demonstrating that a mild aromatic diazonium salt treatment does not give rise to an obvious change in the surface morphology of modified fibers. This may be due to the nanosize thickness of the introduced phenol groups. In contrast, the surface of CF-cardanol (Figure 4c) became much rougher compared to those of untreated CF and CF-OH. The homogeneous dispersion of the modified molecules in various directions could significantly change the surface microstructures, thereby effectively increasing the fiber surface roughness. As a result, the composites interfacial strength was be improved owing to the improvement in the mechanical interlocking effect and physical entanglement density with the matrix at the interface due to the increased roughness. The introduced cardanol could connect CFs and the matrix via the formed covalent bonding between CFs and the matrix, leading to the significant enhancement of the composite’s interfacial and mechanical properties.

### 3.3. Surface Wettability Analysis of CFs

The surface wettability and interfacial compatibility of CFs with the hot matrix are key factors for the interfacial properties of composites. The increased polar groups and the improved surface roughness are beneficial to the fiber surface energy, and thus lead to the enhancement of interfacial wettability. Hence, the contact angle (*θ*) and the surface energy (γ) were evaluated and are summarized in Table 2. The untreated CF had a relatively low surface energy of 35.86 mN·m^−1^ owing to the inert fiber and smooth graphite surface, confirming the poor interfacial compatibility between CFs and the matrix resin. Compared to the untreated CF, the contact angles of CF-OH decreased from 78.50° to 65.14° in polar water, as well as from 58.91° to 54.63° in diiodomethane. As a result, its surface energy, the dispersion component (γ*^d^*), and the polar component (γ*^p^*) improved to 44.34, 31.66, and 12.68 mN·m^−1^, respectively. This may have been due to the many introduced polar phenolic hydroxyl groups. For CF-cardanol, the obvious decrease of polar phenolic hydroxyl groups modified onto the fiber surface resulted in a decrease in the polar component (9.40 mN·m^−1^) compared to that of CF-OH. However, the surface microstructures and morphologies of the fibers changed a lot after the HCCP and cardanol copolymerization. Hence, the dispersion component of CF-cardanol increased sharply to 38.13 mN·m^−1^, which is attributed to the improved roughness and the differences of graphite structures between CFs and the modified molecules. The CF-cardanol sample showed the highest total surface energy with 47.53 mN·m^−1^. The increased surface energy helped to improve the wettability and interfacial compatibility between CFs and the hot resin, which contributed to the composite’s interfacial improvement.

### 3.4. Interfacial Property Testing of Composites

**Figure 5:**
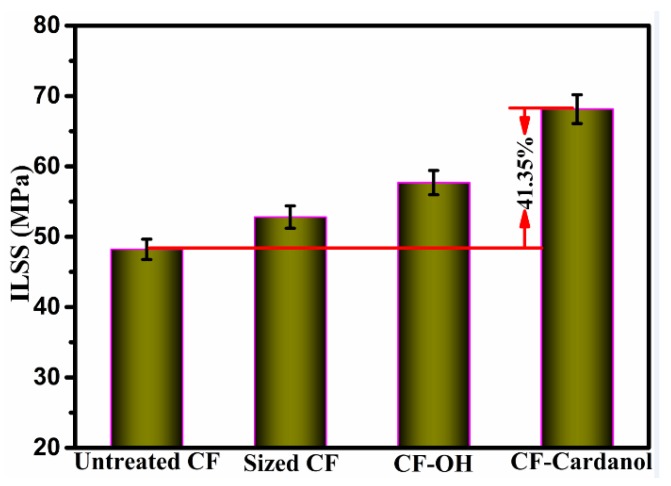
ILSS results of untreated and modified composites.

The overall performance of composites depends mainly on the interfacial adhesion between CFs and the matrix resin. The ILSS values of the untreated CF, sized CF, CF-OH, and CF-cardanol composites are shown in Figure 5. The ILSS value of the UPR composites reinforced with sized CF was 52.79 MPa owing to the commercial polymer sizing layer onto the fiber surface. The ILSS value of the untreated CF composites was only 48.2 MPa. After the aryl diazonium treatment, the CF-OH composites showed an ILSS value of 57.68 MPa, which was a 19.67% improvement compared to that of untreated CF composites owing to the good compatibility and the high activity of the phenolic hydroxyl group with UPR. The ILSS value of CF-cardanol composites was 68.13 MPa, with an increase of 41.35% compared to that of the untreated CF composites and 18.11% compared with that of the CF-OH composites. On one hand, the created hierarchical structure with a high surface roughness formed a gradient modulus at the interface, which could enhance the mechanical interlocking points with the hot resin. On the other hand, the HCCP/cardanol layers increased the fiber surface energy dramatically, and thus enhanced the interfacial wettability between the CFs and matrix significantly. More importantly, an unsaturated C15 hydrocarbon chain of CF-cardanol used as the agents can be easily reacted with the hot resin to form cross-linked structures during the thermal curing process. In addition, comparing the ILSS values of the sized CF and CF-cardanol composites, the sized CF composites had a lower ILSS value compared with that of CF-cardanol composites, indicating that the modification method of cardanol onto the fiber surface was able to enhance the interfacial properties of composites, and showcase the novelty and importance of the work. Hence, the ILSS value of CF-cardanol composites was significantly improved.

**Figure 6:**
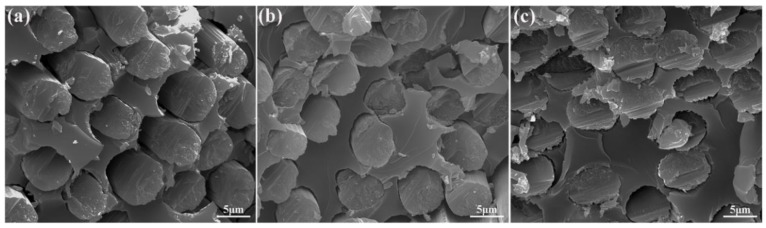
SEM morphologies of the fracture surface of composites reinforced with (**a**) untreated CF, (**b**) CF-OH, and (**c**) CF-cardanol.

To further directly study the reason for the improved ILSS values of the CF-cardanol composites, SEM images showing different composites fracture surfaces are given in Figure 6. There were many holes on the fracture surfaces of the untreated CF composites (Figure 6a) because of massive pulled-out fibers from the matrix resin and the obvious formation of interface debondings, indicating the poor quality of the fiber–matrix interface. The fracture surface of CF-OH/UPR composites (Figure 6b) after ILSS testing was covered with a large amount of fragmented resin. The existence of holes, pulled-out CFs, and interface debondings were much less than those of untreated composites. As for CF-cardanol composites (Figure 6c), there was no interface debonding, fracture step, or pulled-out fibers on the fracture surfaces, confirming the formation of a strong interfacial adhesion between CF-cardanol and UPR.

### 3.5. Hydrothermal Aging Resistance Testing of Composites

**Figure 7:**
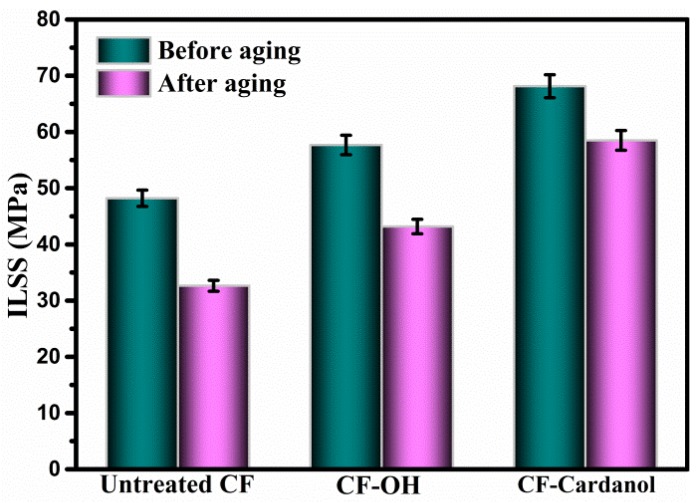
ILSS of composites before and after hydrothermal aging treatment.

As engineering structural materials, carbon-fiber-based polymer composites are widely used in harsh environments with high temperatures and humidity. The composites absorb moisture easily, which can destroy the fiber–matrix interface. Hence, it severely affects the long-term durability and performances of the resultant composites. Therefore, higher requirements on the anti-hydrothermal ageing behaviors of the resulting composites after cardanol grafting have been put forward. The ILSS values of UPR composites reinforced with untreated CF, CF-OH, and CF-cardanol composites before and after hydrothermal aging are shown in Figure 7. After aging, the ILSS value of untreated CF composites decreased to 36.23 MPa from an ILSS value of 48.2 MPa without aging with an ILSS retention ratio of only 75.17%. Water uptake was usually transported via micro-cracks in the composites. Voids, defects, and microcracks were easily formed at the fiber–matrix interface arising from the weak interfacial quality of the untreated CF-UPR interface. Under the hydrothermal aging condition, many water molecules penetrated into the interface region easily through these formed microcracks or voids, and thus deteriorated the matrix resin and fiber–matrix interface, leading to the poor anti-hydrothermal ageing behaviors of the untreated CF composites. The ILSS of CF-OH composite after aging was 43.17 MPa, and the ILSS retention ratio of composites was about 74.84%. However, the ILSS value of the UPR composites reinforced with CF-cardanol composites after 48 h of hydrothermal aging was 58.49 MPa with a slightldecrease of 14.15% compared with those of the untreated CF and CF-OH composites. The introduced hierarchical HCCP/cardanol copolymerization layers at the interface acting as the buffer layer could inhibit microcrack formation, and then prevent water molecules from damaging the interface effectively. Moreover, the best quality of the interface among the three fibers was to be destroyed by needing more energy and longer time. Therefore, the hydrothermal aging resistance of CF-cardanol composites is obviously improved.

## 4. Conclusions

In this study, carbon fibers functionalized with renewable cardanol were successfully synthesized via a facile in situ polymerization with the aim of changing the fiber surface microstructures and improving the interfacial properties of the UPR composites. Massive modified cardanol molecules produced dramatically enhanced interfacial wettability and compatibility between the CFs and UPR, and also created chemical bonding at the interface through the cross-linked reaction between cardanol and UPR, leading to a higher-quality interface. As a result, the interfacial adhesion of CF-cardanol composites was enhanced significantly, affording an ILSS value (68.13 MPa) that was an increase of 41.35% compared to that of untreated CF composites (48.2 MPa). Moreover, the best quality of the interface by introducing renewable cardanol structures was that it could protect the interface effectively under hydrothermal aging conditions, resulting in the best anti-hydrothermal ageing behaviors among the three types of fibers.

## Figures and Tables

**Table 1:** Surface element analysis of the carbon fibers (CFs).

Samples	Element Content (%)					
	C	N	O	P	Cl	O/C
Untreated CF	81.61	0.92	17.47	–	0.21	0.21
CF-OH	75.40	1.21	23.39	–	0.33	0.31
CF-Cardanol	70.48	6.49	18.35	3.61	1.07	0.26

**Table 2:** Contact angles, surface energy, and the integral area ratio of D to G of different CFs.

Samples	Contact Angles (°)		Surface Energy (mN∙m^−1^)			*R, I_D_/I_G_*
	θ_water_	θ_diiodomethane_	γ*^d^*	γ*^p^*	γ	
Untreated CF	78.50	58.91	29.20	6.66	35.86	3.49
CF-OH	65.14	54.63	31.66	12.68	44.34	3.84
CF-Cardanol	66.82	42.89	38.13	9.40	47.53	3.83
